# Evaluating the Clinical Utility of Brachial Plexus Block for Reducing Opioid Exposure in Pediatric Elbow Fracture Surgery: A Retrospective Cohort Study

**DOI:** 10.3390/medicina60030483

**Published:** 2024-03-14

**Authors:** Kunhyung Bae, Yeon Ju Kim, Hyo Won Lim, Michael Seougcheol Kang, Ha-Jung Kim, Won Uk Koh, Young-jin Ro, Jooyeon Cho, Hwa Jung Kim, Soo-Sung Park, Yoon Hae Kwak, Hyungtae Kim

**Affiliations:** 1 Department of Orthopedic Surgery, Hanyang University Hospital, Hanyang University College of Medicine, 222 Wangsimni-ro, Seongdong-gu, Seoul 04763, Republic of Korea; bae_k_h@naver.com (K.B.); hyowon1201@gmail.com (H.W.L.); 2 Department of Anesthesiology and Pain Medicine, Asan Medical Center, University of Ulsan College of Medicine, 88, Olympic-ro, 43-gil, Songpa-gu, Seoul 05505, Republic of Korea; yjans@amc.seoul.kr (Y.J.K.); d100262@amc.seoul.kr (H.-J.K.); wu.koh@amc.seoul.kr (W.U.K.); yjro@amc.seoul.kr (Y.-j.R.); cho.chris197@gmail.com (J.C.); 3 Department of Orthopedic Surgery, Asan Medical Center Children’s Hospital, University of Ulsan College of Medicine, 88, Olympic-ro, 43-gil, Songpa-gu, Seoul 05505, Republic of Korea; kang@amc.seou.kr (M.S.K.); sspark@amc.seoul.kr (S.-S.P.); 4 Department of Clinical Epidemiology and Biostatistics, Asan Medical Center, University of Ulsan College of Medicine, Olympic-ro, 43-gil, Songpa-gu, Seoul 05505, Republic of Korea; rsvp@amc.seoul.kr

**Keywords:** analgesics, opioid, brachial plexus block, elbow fractures, nerve block, pain management, pediatrics, retrospective studies

## Abstract

*Background and Objectives*: Reducing opioid exposure in common pediatric surgeries is of paramount importance. This study aimed to assess the efficacy of regional nerve blocks in reducing opioid exposure while preserving high success rates. *Materials and Methods*: We conducted a retrospective matched cohort study (1:1) including patients with elbow fractures < 12 years old who underwent treatment with percutaneous pinning. Patients were divided into general-anesthesia (GA) and GA-followed-by-supraclavicular-brachial-plexus-block (GA-SCB) groups. The primary outcome was the number of patients administered postoperative rescue opioids. The secondary outcomes included intraoperative and postoperative opioid administration, the time to first request for rescue analgesia, pain scores, block success rate, block performing time, and block-related complications. *Results*: In a total of 478 patients, 363 underwent percutaneous pinning, and 86 were cohort-matched (GA: *n* = 43, GA-SCB: *n* = 43). On the first postoperative day, 34 (79.0%) patients in the GA group were administered postoperative rescue opioids, compared with 12 (27.9%) in the GA-SCB group (*p* < 0.001). All the patients in the GA-SCB group were opioid-free during the intraoperative period. No SCB-associated complications were observed. Total opioid consumption was significantly lower in the GA-SCB group than in the GA group until the first postoperative day (GA vs. GA-SCB, 3.2 ± 3.0 mg vs. 0.9 ± 1.8 mg, *p* < 0.001). *Conclusions*: SCB application in pediatric patients who underwent elbow fracture surgery significantly reduced opioid exposure and had a high success rate when performed using ultrasound guidance by an expert. Furthermore, the complication risk and surgical delay were minimal.

## 1. Introduction

An elbow fracture is one of the most common pediatric orthopedic traumas, accounting for 15–20% of all pediatric fractures [1]. The most common types of elbow fracture are supracondylar humerus fractures (SCHF), followed by lateral condylar fractures (LCF), and radial neck fractures (RNF). In children, these can be treated with closed reduction and percutaneous pinning (CRPP), if surgical treatment is indicated [2,3]. In pediatric elbow fractures with CRPP, intravenous opioid analgesics are commonly used for acute postoperative management of moderate to severe pain [4]. However, there is a paucity of literature on postoperative pain management after pediatric elbow fracture surgery [5], and the need for regional nerve blocks in this setting remains controversial. Regional nerve blocks in pediatric patients may be underutilized because of concerns regarding potential complications associated with the block, the belief that children’s pain perception is underdeveloped, and the highly demanding nature of the block, which requires time and human resources [6,7].

In the case of pediatric elbow fracture surgery, numerous institutions favor opioid administration as opposed to the utilization of regional nerve blocks, thus contributing to the issue of opioid overuse [4]. Exposure of pediatric patients to opioids could increase the adverse events associated with opioid use and increase the likelihood of subsequent misuse, even when opioid exposure remains within legal bounds in pediatrics and adolescents [8]. However, as per the data provided by the Pediatric Regional Anesthesia Network, it is evident that few institutions routinely perform upper limb nerve block [9]. Thus, anesthesiologists and orthopedic surgeons must develop a thorough understanding of the regional nerve block, common procedures in pediatric patients, and the patterns of opioid use before and after regional nerve block application. In addition, delving into the complication rate associated with regional nerve blocks and assessing their impact on operative time would be of significant benefit to anesthesiologists and surgeons.

In the past, our hospital traditionally relied on opioids for active pain management in pediatric surgery. Given the emergence of the opioid crisis as a significant social issue globally, we have proactively incorporated regional nerve blocks in pediatric patients since 2022. The purpose of this study was to evaluate whether regional nerve blocks can effectively decrease opioid exposure, maintain high success rates, and avoid an increase in complication rates or significant surgical delays.

## 2. Materials and Methods

### 2.1. Study Design and Patients

This was a retrospective study. The study was conducted following the STROBE guidelines [10], approved by the Asan Medical Center Institutional Review Board (No. 2023-0427). We reviewed the electronic medical records of patients with elbow fractures between April 2015 and March 2023. This study was conducted at the Department of Orthopedic Surgery of Asan Medical Center, a tertiary hospital.

The inclusion criteria were patients < 12 years old who underwent CRPP for an elbow fracture as surgical treatment. The exclusion criteria were the following: (i) surgery on any other part of the body during hospitalization, (ii) past history of elbow surgery, (iii) underlying baseline medical conditions requiring regular pain management, (iv) open fractures, and (v) multiple fractures. Patients who met the inclusion criteria were grouped based on the anesthetic method followed, i.e., general anesthesia (GA) and GA-followed-by-supraclavicular-brachial-plexus-block (GA-SCB). At our hospital, GA was performed for pediatric elbow fracture patients until May 2022, and the GA-followed-by-SCB was performed since June 2022. Therefore, patients who underwent GA were matched in a 1:1 ratio with patients from the GA-SCB group for sex, age, and diagnosis. The most recent GA patient was selected when two or more candidates were available. For this study, the elbow fractures for which CRPP was performed were supracondylar humerus fracture (SCHF), lateral condylar fracture (LCF), and radial neck fracture (RNF). No patients needed tourniquet during procedures due to CRPP.

### 2.2. Anesthesia Technique

For the GA group, patients were placed in the supine position on standard monitoring procedures, including the use of non-invasive blood pressure monitoring, an electrocardiogram, and a pulse oximeter. Thiopental sodium (4–5 mg/kg) was injected intravenously. After the patients lost consciousness, the lungs were mask ventilated with 6–7 vol% sevoflurane in 80% oxygen for 3 min, and an Ambu^®^ AuraGain™ (Ambu, Ballerup, Denmark) of appropriate size based on the body weight of the patient was inserted without a neuromuscular blocking agent. All SCBs were performed by the same regional anesthesiologist (HK) after the induction of GA. A high-frequency linear transducer (5–18 MHz, SONIMAGE HS1, Konica Minolta, Tokoy, Japan) was used for SCB. In all cases, 0.2% ropivacaine up to 1 mL/kg (2 mg/kg) was used. The ultrasound probe was placed under sterile conditions on the supraclavicular fossa to obtain a short-axis view of the subclavian artery and the neural cluster (trunks/divisions of the brachial plexus). Using an in-plane technique and a lateral to medial direction, the blocking needle was advanced until its tip was positioned at the “corner pocket”. Half of the local anesthetic was deposited at this location. The needle tip was then repositioned posterolaterally to the neural cluster. The remaining volume was injected at this site. The surgical procedure was performed after anesthesia was introduced. GA was maintained with sevoflurane in 40% oxygen, and the end-tidal carbon dioxide level was maintained in the range of 35 ± 5 mmHg. The concentration of sevoflurane was adjusted in the range of 2–3 vol% according to the bispectral index and vital signs. Intravenous fentanyl (0.5 µg/kg) was administered during the intraoperative period when a rapid increase in baseline heart rate or arterial pressure of ≥20% was monitored.

### 2.3. Postoperative Care and Pain Management of Elbow Fracture

After CRPP of pediatric elbow fractures, radiographs were taken to confirm the anatomical reduction in the operating room. Then, patients were transferred to the post-anesthesia care unit (PACU). In the PACU, pain was assessed on arrival and discharge using the Face, Legs, Activity, Cry, Consolability (FLACC) scale. Intravenous fentanyl (0.5 µg/kg) was administered when the pain score exceeded 4. In the ward, pain was assessed on arrival, on the first morning after surgery, and whenever patients complained of pain. Pain was assessed in the ward using FLACC for patients aged < 8 years and the numerical rating scale for patients aged ≥ 8 years. Intravenous ibuprofen (10 mg/kg) was the first option for pain relief and was administered if patients asked for analgesics or experienced pain with a pain score exceeding 4. The pain score was re-evaluated after the first ibuprofen (Caldolor^®^, DB Pharm Korea Co., Seoul, Republic of Korea) injection. If the pain score exceeded 4 after 1 h of ibuprofen injection, then intravenous pethidine (1 mg/kg) was added for pain relief. The patients were admitted to the hospital for a period of one to two days for post-operative care. All of the patients were placed in a long-arm cast for a period of four to five weeks. One month after the operation, the bone union was assessed in the outpatient clinic and the K-wire was removed under general anesthesia.

### 2.4. Investigated Variables

The primary outcome was the number of patients who received postoperative rescue opioids. Secondary outcomes included intraoperative opioid consumption, postoperative opioid consumption during the postoperative 24 h, the time to first request for rescue analgesia, and FLACC scores during the postoperative 24 h. In addition, block success rate, block performing time, and block-related complications were investigated. Opioid consumption was converted to morphine milligram equivalents. The following time points of the FLACC score were investigated: (i) on arrival at the PACU (postoperative 0–1 h), (ii) on discharge from the PACU (postoperative 1–2 h), (iii) on arrival at the ward (postoperative 2–4 h), (iv) postoperative 12 ± 2 h, and (v) first postoperative day (postoperative 24 ± 2 h). The success rate of the block was determined by evaluating cases for whom intravenous opioid (fentanyl) was administered due to elevated heart rate or arterial pressure during the intraoperative phase as instances of block failure [11]. Preoperative anesthesia duration was defined as the time from the induction of general anesthesia to the start of the surgical procedure. We opted to define the discrepancy in time between the two groups as the block-performing time. Block-related complications included paresthesia, motor deficit, and hematoma or pneumothorax until the first postoperative day.

### 2.5. Statistical Analysis

Student’s *t*-test and chi-squared test were used to assess significant differences between demographic characteristics, pain scores, and opioid consumption. Logistic regression models were used to determine whether baseline characteristics would affect opioid administration during the first postoperative day. Opioid-free duration (i.e., in the case of patients who received opioids for different durations of time) was compared between the GA and GA-SCB groups using Kaplan–Meier curves and log-rank tests. 

The statistical analyses were performed using SAS software (version 9.4, SAS Institute, Cary, NC, USA). *p*-values < 0.05 were considered statistically significant.

## 3. Results

**Figure 1:**
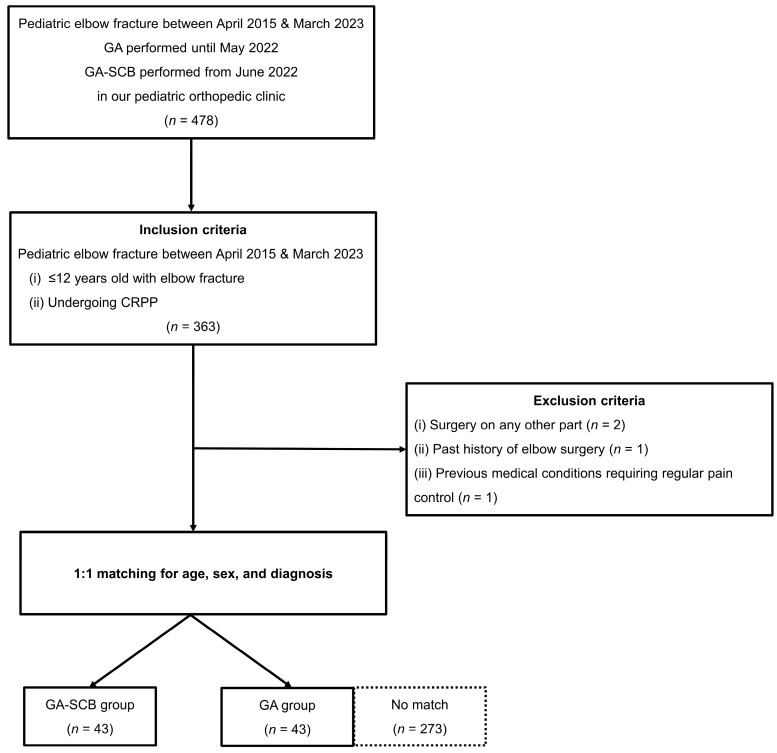
Flowchart of the study. GA, general anesthesia; GA-SCB, general anesthesia-supraclavicular brachial plexus block; CRPP, closed reduction and percutaneous pinning.

Approximately 478 pediatric elbow fracture patients were enrolled between April 2015 and March 2023. Of these, patients were given GA until May 2022 and SCB with GA from June 2022. Of these, 363 patients < 12 years old with elbow fractures who underwent CRPP were included in the cohort. We performed 1:1 cohort matching by age, sex, and diagnosis in all patients, except for four patients who met the exclusion criteria. After that, 36 patients were finally analyzed (Figure 1).

A total of 43 patients who met the criteria received GA with SCB after June 2022, resulting in 43 matched individuals allocated to the GA group. The baseline demographic characteristics showed no significant difference between the two groups (Table 1).

**Figure 2:**
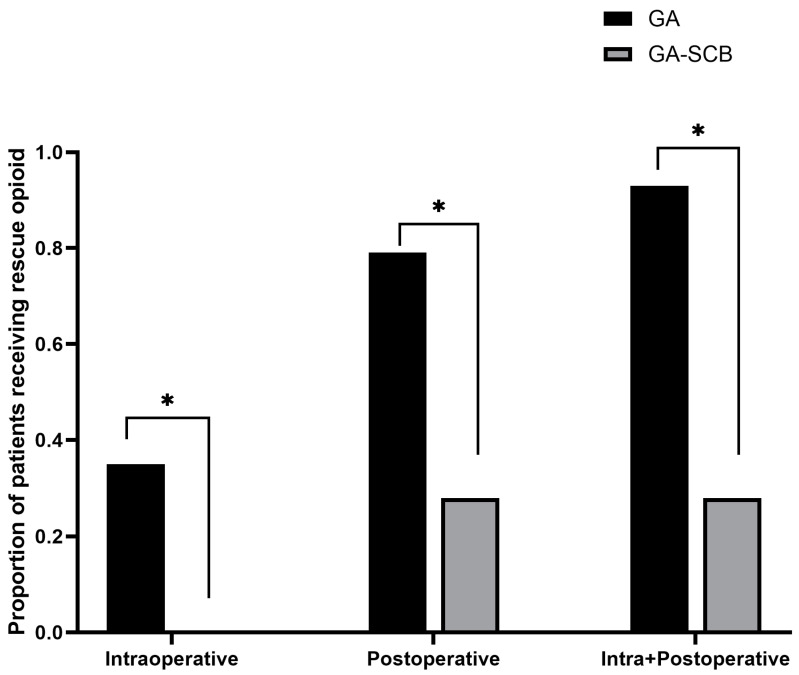
Proportion of patients that were administered rescue opioids (* *p* < 0.001). GA, general anesthesia; GA-SCB, general anesthesia-supraclavicular brachial plexus block.

The number of patients who received postoperative rescue opioids was 34 (79.0%) in group GA and 12 (27.9%) in group GA-SCB on the first postoperative day (*p* < 0.001) (Figure 2).

All patients in the group GA-SCB achieved opioid-free conditions during the intraoperative period. Considering this, it was reasonable to categorize the block success rate as 100%. In addition, in the logistic regression analysis, the anesthetic method was the only significantly associated predisposing factor for postoperative opioid consumption (odds ratio [OR], 0.013; 95% CI, 0.002–0.077; *p* < 0.001) (Table 2).

Total opioid consumption was significantly lower for patients in the GA-SCB group than those in the GA group during the intraoperative period (GA vs. GA-SCB, 0.8 ± 1.3 mg vs. 0 mg, *p* < 0.001) and until the first postoperative day (GA vs. GA-SCB, 3.2 ± 3.0 mg vs. 0.9 ± 1.8 mg, *p* < 0.001). Patients in the GA-SCB group had significantly lower FLACC scores at arrival in PACU, discharge from PACU, and the first pain check in the ward (GA vs. GA-SCB: 4.9 ± 2.3 vs. 0.9 ± 1.5, *p* < 0.001; 3.1 ± 2.5 vs. 0.9 ± 1.5, *p* = 0.143; 2.2 ± 1.8 vs. 1.0 ± 1.4, *p* = 0.017, respectively) (Table 3).

The most severe postoperative pain was greater for patients in the GA group, although this was not statistically significant (pain score: GA vs. GA-SCB; 5.4 ± 2.0 vs. 4.7 ± 2.1, *p* = 0.143). Patients in the GA group had severe pain immediately after surgery, which then decreased gradually. Concurrently, patients in the GA-SCB group had less pain after surgery until 12 h, after which the pain increased significantly. 

**Figure 3:**
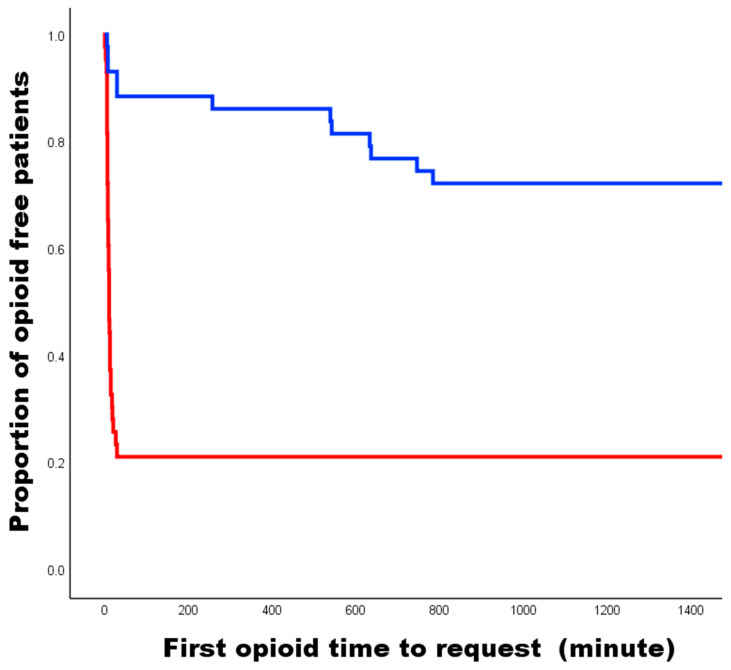
Kaplan–Meyer graph of postoperative first opioid time-to-patient request. The red line indicates the GA group and the blue indicates the GA-SCB group. GA, general anesthesia; GA-SCB, general anesthesia-supraclavicular brachial plexus block.

The first opioid time-to-patient request was significantly faster for patients in the GA group than those in the GA-SCB group (GA vs. GA-SCB, 10 ± 6.3 min vs. 351 ± 323.1 min, *p* < 0.001). Specifically, in the GA-SCB group, opioids were initially administered in the mid-postoperative period according to patient request, whereas in the GA group, opioids were administered in the early period (Figure 3). 

No SCB-associated complications were observed. Although the preoperative anesthesia duration was significantly shorter for patients in the GA group than for those in the GA-SCB group, the difference (considered block performing time) was only 9.2 min (GA vs. GA-SCB, 21.1 ± 11.2 min vs. 30.3 ± 7.7 min, *p* < 0.001).

## 4. Discussion

In this retrospective study, we demonstrated that the application of SCB in pediatric elbow fracture surgery significantly lowered the rate of postoperative opioid exposure (from 79.0% to 27.9%) with a high success rate (achieving an intraoperative opioid administration rate of 0%). In addition, no complications were observed, and the impact on surgical duration was minimal, with only a 9.2-min delay.

Elbow fractures are a common cause of pediatric orthopedic trauma, accounting for 10–15% of pediatric fractures, followed by wrist and hand fractures [1,12]. The peak incidence of elbow fractures is in children between 5 and 10 years of age and is predominant in boys [13]. The most common type is the SCHF, which accounts for approximately 60% of cases and is usually caused by falling on an outstretched hand, resulting in hyperextension of the elbow [14]. These fractures are classified according to the degree of displacement, using the Gartland classification [3]. Another significant fracture is LCH, which accounts for approximately 15 to 20% of fractures [15]. Moreover, it is usually caused by a varus force to the elbow, resulting in avulsion on the lateral aspect of the elbow condyle, and is classified using the Milch or Jakob classification system [16]. RNF is the third most common type of pediatric elbow fracture and often occurs when a child falls onto an outstretched hand, with a valgus force to the radial neck [12]. The correct diagnosis and treatment should be personalized for each patient to avoid malunions or deformities, such as cubitus varus or valgus [17]. They are diagnosed by clinical assessment with radiological imaging, including simple radiographs or CT scans, to determine the type of fracture and assess associated injuries [12]. Conservative treatment, such as closed reduction and immobilization, is usually sufficient for minimal fractures, whereas more displaced cases may require CRPP if surgical treatment is required [2,3]. CRPP is a relatively standardized surgical technique for the treatment of pediatric elbow fractures and has shown favorable postoperative results [18]. If the same surgical technique (CRPP) is used to target the same site of trauma (the elbow), the perioperative pain among these patients would be comparable. With regard to other factors such as patient position, Sapienza M. et al recently reported no difference between supine and prone position, so we included all patients with CRPP regardless of patient position [19].

Inadequate management of postoperative pain adversely affects pediatric patients and can lead to exaggerated responses to pain in adulthood [20]. As a result, the need for active pain management in the postoperative period has increased in pediatric surgery. Conventional postoperative pain management relies on opioid analgesics, even in the pediatric setting. However, the adverse effect profile in children has been recognized, including acute opioid-induced respiratory depression or tolerance to opioid analgesic effects [21,22]. Alternatives have been proposed to reduce opioid use while maintaining adequate pain control, including the use of peripheral nerve blocks. The 2018 Committee of the Pediatric Orthopedic Society of North America suggested that alternative interventions such as increased physician and patient education, prescription monitoring and drug disposal programs, peripheral nerve block, and non-opioid analgesic alternatives are needed for improving opioid stewardship in pediatric orthopedic surgery [6,23]. Therefore, opioid-free analgesia, in addition to reduced opioid use, is being pursued in common pediatric trauma.

Opioid administration is commonly used for managing acute pain following surgical procedures [24]. Nevertheless, the extent of opioid administration varies significantly and is influenced by institutional policies and surgeon preferences, contributing to excessive opioid administration [25]. In the context of pediatric elbow fracture surgery, a spectrum of practices exists, ranging from underestimating the severity of pain to the overprescription of opioids. Our study evaluated the effect of SCB on opioid exposure and the dose used in pediatric elbow fracture surgery. Our study showed that before the application of SCB, 79% of patients had postoperative opioid exposure. This number increased to 93% when intraoperative opioid exposure was included, indicating a substantial exposure to opioids among most patients. However, after the introduction of SCB, there was a significant reduction to 27.9%, considering both intra- and postoperative opioid use. This reduction in opioid exposure has significant importance, particularly considering the age profile of the patients involved.

Pediatric patients possess smaller nerves, blood vessels, and other anatomical structures in comparison to adults, potentially diminishing the success rate of regional blocks. In our study, we used rescue opioids in response to alterations in intraoperative vital signs and subsequently determined the block success rate. While the sample size was small in each group (43 per group), it is noteworthy that the success rate was exceptionally high. Our findings aligned with findings from a previous study by Zadrazil M et al., which consisted of 565 cases [11]. The high success rate of the block in our study could be attributed to the fact that it was ultrasound-guided by an experienced practitioner and conducted under general anesthesia conditions, ensuring minimal interference from patient movement.

This study observed that patients in the GA-SCB group had significantly less pain, especially in the early postoperative period. Peripheral nerve block is reported to generate significantly lower pain scores and improve analgesic quality compared with the traditional opioid-based technique [26]. However, there is a problem with rebound pain after SCB caused by the dissolution of the local anesthetic [27,28]. Rebound pain is usually reported to begin 12–24 h after SCB and last for approximately 2 h [29]. In this study, at approximately 12 h postoperatively, pain scores were higher in patients in the GA-SCB group than in those in the GA group. This phenomenon was expected, and even the most severe pain was tolerable and well-controlled [30]. Considering that pediatric patients who underwent elbow fracture surgery were discharged on the first postoperative day, additional use of oral analgesics and aggressive use of regional nerve block would be beneficial.

None of the 43 patients in the GA-SCB group experienced block-related complications. Previously, there had been concern about peripheral nerve block in anesthetized patients because of the risk for neurovascular injury or iatrogenic pneumothorax [31], but recent studies have shown no increased risk with this approach [32,33]. Real-time imaging of the associated neurovascular structure enables precise needle placement, reducing the amount of anesthetic required and limiting the risk of anesthetic toxicity. With these technological advances, pediatric regional anesthesia can be performed safely with minimal risk for neurological injury [34]. In a retrospective review of 1657 peripheral nerve blocks performed in pediatric patients after induction of general anesthesia, only two self-limiting nerve injuries were reported [35]. Similarly, in our study, all pediatric patients underwent SCB under general anesthesia. Based on the findings of the Pediatric Regional Anesthesia Network, it is recommended that regional blocks can and should preferably be performed under general anesthesia or deep sedation in children of all ages.

Nevertheless, the peripheral nerve block may be controversial in terms of cost-effectiveness. Additionally, it will be less effective when the peripheral nerve block duration is relatively longer than the surgery duration. In this study, the preoperative anesthesia duration was significantly shorter in the GA-SCB group compared to that in the GA group. However, for experts, this difference in duration is likely to be less than 10 min. Considering the quality of postoperative recovery and the reduction in opioid consumption, this time duration should be acceptable.

The study has several limitations. Our study has an inherent limitation because of its case-control design. The methodology was retrospective and not double-blinded; however, patients and related healthcare providers were not informed that a study was being conducted. Therefore, opioid administration and patient pain assessment were not subjected to external bias. Second, the assessment of postoperative rebound pain in the GA-SCB group may not be accurate. There appears to be a slow increase in rebound pain, followed by a decrease in the GA-SCB group. The lack of a detailed assessment of pain timing did not reflect the true mirror image of the pain graph. However, owing to the lack of assessment in the intervening period, pain scores rapidly increased as the regional anesthesia resolved and rapidly decreased as the opioid analgesic was administered. Third, the included patients were all elbow trauma only with CRPP; therefore, further studies are warranted investigating other types of traumas with different operative methods.

## 5. Conclusions

In conclusion, the application of SCB in pediatric patients who underwent elbow fracture surgery significantly reduced opioid exposure and had a high success rate when performed by an expert hand using ultrasound guidance. Furthermore, the complication risk or surgical delay that could prevent clinicians from adopting regional anesthesia is minimal.

## Figures and Tables

**Table 1:** Patient baseline characteristics.

Characteristics	GA (*n* = 43)	GA-SCB (*n* = 43)	*p*-Value
Age, years	6.9 ± 2.4	6.8 ± 2.4	0.634
BMI, kg/m^2^	17.0 ± 2.6	16.9 ± 3.2	0.795
Gender, Male	19 (44.1)	19 (44.1)	1.000
Diagnosis			1.000
SCHF	32 (74.4)	32 (74.4)	
LCF	6 (14.0)	6 (14.0)	
RNF	5 (11.6)	5 (11.6)	

Results are expressed as mean ± SD, *n* (%). GA, general anesthesia; GA-SCB, general anesthesia followed by supraclavicular brachial plexus block; BMI, body mass index; SCHF, supracondylar humerus fracture; LCF, lateral condylar fracture; RNF, radial neck fracture.

**Table 2:** Univariate logistic regression analysis for postoperative opioid consumption until the first postoperative day.

Characteristics	OR	95% CI	*p*-Value
Age	0.892	0.638–1.247	0.505
Sex: Male	2.745	0.697–10.814	0.149
Diagnosis			0.560
SCHF (reference)			0.911
LCF	0.878	0.090–8.599	0.430
RNF	0.319	0.019–5.438	0.125
BMI	0.797	0.596–1.065	0.126
Anesthetic methods: GA-SCB	0.013	0.002–0.077	<0.001

OR, Odds Ratio; CI, confidence interval; SCHF, supracondylar humerus fracture; LCF, lateral condylar fracture; RNF, radial neck fracture; BMI, body mass index; GA-SCB, general anesthesia followed by supraclavicular brachial plexus block.

**Table 3:** Perioperative opioid consumption and postoperative pain score.

Characteristics	GA (*n* = 43)	GA-SCB (*n* = 43)	*p*-Value
Opioid consumption			
Intraoperative ^a^	0.8 ± 1.3	0	<0.001
During postoperative 24 h ^a^	3.2 ± 3.0	0.9 ± 1.8	<0.001
FLACC scale ^b^			
Arrival in PACU (postoperative 0–1 h)	4.9 ± 2.3	0.9 ±1.5	<0.001
Discharge from PACU (postoperative 1–2 h)	3.1 ± 2.5	0.4 ± 0.8	<0.001
First pain check in the ward (postoperative 4 ± 2 h)	2.2 ± 1.8	1.0 ± 1.4	0.017
Pain check at postoperative 12 ± 2 h	1.5 ± 1.5	3.8 ± 2.4	<0.001
POD 1 day (postoperative 24 ± 2 h)	1.0 ± 1.5	0.8 ± 0.9	0.36
Worst pain until POD 1	5.4 ± 2.0	4.7 ± 2.1	0.143

Results are expressed as mean ± SD. GA, general anesthesia; GA-SCB, general anesthesia followed by supraclavicular brachial plexus block; FLACC scale, Face, Legs, Activity, Cry, Consolability scale; PACU, post-anesthesia care unit; POD, postoperative day. ^a^ Morphine milligram equivalent (mean ± SD). ^b^ Pain score, FLACC scale for patients aged < 8 years, and the numerical rating scale for patients ≥ 8 years.

## Data Availability

The dataset used and/or analyzed during the current study is available from the corresponding author upon reasonable request.
